# Prognostic roles of signal transducers and activators of transcription family in human breast cancer

**DOI:** 10.1042/BSR20171175

**Published:** 2018-12-11

**Authors:** Shuntao Wang, Lili Yu, Wei Shi, Xueqin Li, Lixiu Yu

**Affiliations:** 1 Department of Breast and Thyroid Surgery, Union Hospital, Tongji Medical College, Huazhong University of Science and Technology, Wuhan 430022, China; 2 Department of Obstetrics and Gynecology, Union Hospital, Tongji Medical College, Huazhong University of Science and Technology, Wuhan 430022, China; 3 Department of Pharmacy, Union Hospital, Tongji Medical College, Huazhong University of Science and Technology, Wuhan 430022, China

**Keywords:** breast cancer, overall survival, post-progression survival, progression-free survival, signal transducers activators of transcription

## Abstract

Signal transducers and activators of transcription (STAT) family are critical transcription factors, which have been proved as prognostic predictors for a number of cancers. However, the prognostic roles of STAT family in breast cancer patients remain in dispute. In the present study, we mined the ‘Kaplan–Meier plotter’ (KM plotter) online database to explore the prognostic roles of STAT family mRNA expression in breast cancer including overall survival (OS), progression-free survival (PFS), as well as post-progression survival (PPS). The results suggest high mRNA expression of all the individual STATs, except STAT1 and STAT2, are significantly associated with favorable OS in breast cancer patients; high *STAT1* mRNA expression is significantly associated with worse RFS and all the other individual STATs, except STAT3, are significantly associated with better RFS in breast cancer patients; only high *STAT5b* mRNA expression is significantly related to better PPS in breast cancer patients. Additionally, we explored the prognostic values of individual STATs in other clinicopathological features, such as pathological grades, estrogen receptor (ER) status and so on. The results suggest, except STAT2 and STAT6, high mRNA expression of STATs is related to a favorable prognosis especially for high pathological grade; high *STAT5* mRNA expression indicates a favorable prognosis no matter under ER positive or negative status; high *STAT4* mRNA expression suggests a favorable prognosis under human epidermal growth factor receptor 2 (HER2) negative status. Our results indicate that individual STATs, except STAT1 and STAT2, may act as a favorable prognostic biomarker in breast cancer. Nevertheless, further investigations on a larger population are warranted.

## Introduction

Breast cancer is one of the leading causes of cancer deaths amongst women throughout the world, and the number of newly diagnosed is increasing [1]. Despite great efforts to improve early detection and treatment of patients with advances, the mortality of breast cancer is still a global problem [2]. Previous studies have proved that identification and validation of prognostic factors for breast cancer, to a certain extent, could improve clinical outcomes of breast cancer patients [3].

Signal transducers and activators of transcription (STAT) are cytoplasmic transcription factors including a total of seven identified members (STAT1, STAT2, STAT3, STAT4, STAT5a, STAT5b, STAT6) [4]. They are activated mainly by two ways, either by tyrosine kinase receptors after binding of growth factors or by soluble tyrosine kinases of the Janus (JAK) and Src kinase families, after binding of cytokines to their receptors [4]. Tyrosine-pSTATs then dimerize and translocate to the nucleus, where they bind DNA and regulate gene transcription [5,6]. It is proved that STATs can mediate pleiotropic cellular functions including those related to cellular proliferation, survival, and angiogenesis [7,8], and their activation is associated with hormones, cytokines, and growth factors [9,10]. Importantly, previous studies have proved that STATs played a pivotal role in oncogenesis of breast cancer [11–13]. However, the prognostic values of STAT family in breast cancer patients remain in dispute. In the present study, we mined the ‘Kaplan–Meier plotter’ (KM plotter) online database to comprehensively explore the prognostic values of seven STAT genes in breast cancer patients.

## Materials and methods

The prognostic value of *STATs* mRNA expression for breast cancer patients was assessed by using online KM plotter (http://kmplot.com/analysis/index.php?p=service&cancer=ovar) database. Up to now, more than 54000 genes’ effects in several cancers have been evaluated, such as ovarian cancer [14], breast cancer [15], lung cancer [16], and so on. In the present study, we evaluated the overall survival (OS), progression-free survival (PFS), and post-progression survival (PPS) of patients with breast cancer by a Kaplan–Meier survival plot, with the hazard ratio (HR) with 95% confidence intervals (CI) and log-rank *P* value. We only chose the JetSet best probe set of STATs to obtain Kaplan–Meier survival plots. In addition, the clinical data, such as pathological grade, estrogen receptor (ER), human epidermal growth factor receptor 2 (HER2), and tumor protein P53 (TP53) mutation status of breast cancer patients were obtained in this database.

## Results

**Figure 1:**
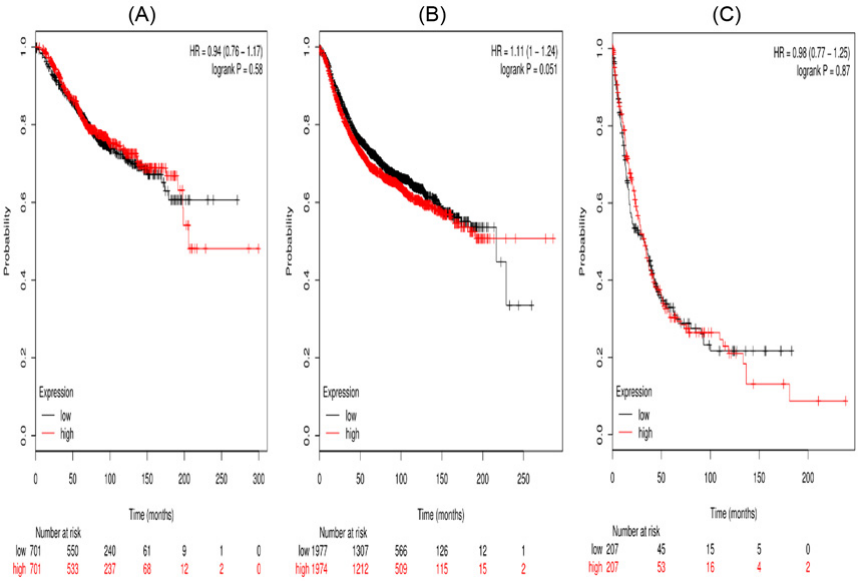
The prognostic value of high *STAT1* mRNA expression in all the breast cancer patients ((**A**) OS curve; (**B**) RFS curve; (**C**) PPS curve)

In the present work, all the seven STAT members’ Kaplan–Meier survival information can be obtained on www.kmplot.com. First, the prognostic value of STAT1 in the database was evaluated. Affymetrix IDs for STAT1:200887_s_at. OS, RFS, and PPS curves were plotted for all the breast cancer patients (*n*=1582), respectively (Figure 1). High mRNA expression of STAT1 was not significantly related to OS (HR =0.94 (0.76–1.17), *P*=0.58) and PPS (HR =0.98 (0.77–1.25), *P*=0.87) in breast cancer patients. While, high mRNA expression of STAT1 was significantly related to worse RFS (HR =1.11 (1–1.24), *P*=0.051).

**Figure 2:**
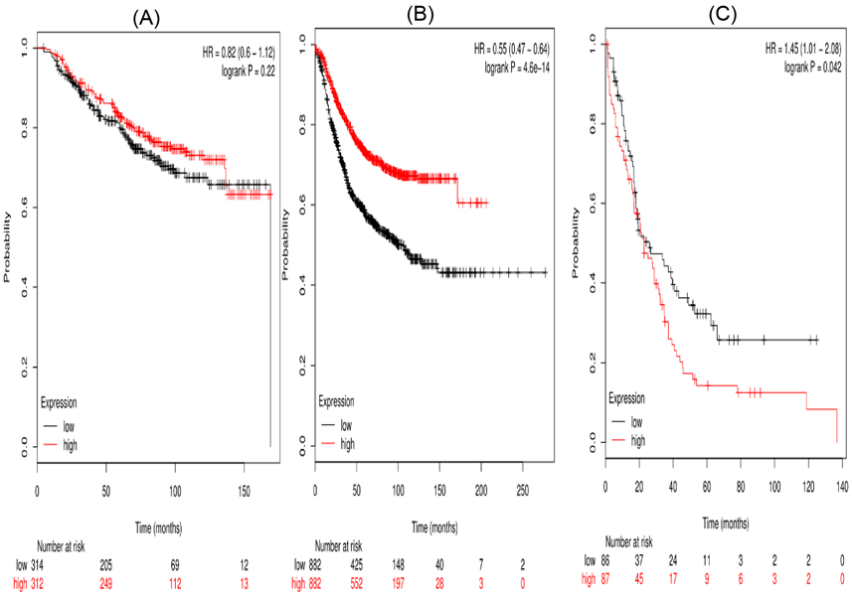
The prognostic value of high STAT2 mRNA expression in breast cancer patients((A) OS curve; (B) RFS curve; (C) PPS curve)

Second, the prognostic significance of *STAT2* mRNA expression in the database was evaluated. Affymetrix IDs for STAT2: 225636_at. High *STAT2* mRNA level showed a null association with OS (HR =0.82 (0.6–1.12), *P*=0.22) in breast cancer patients. However, high mRNA expression of STAT2 was significantly related to better RFS (HR =0.55 (0.47–0.64), *P*<0.001) and worse PPS (HR =1.45 (1.01–2.08), *P*=0.042) (Figure 2).

**Figure 3:**
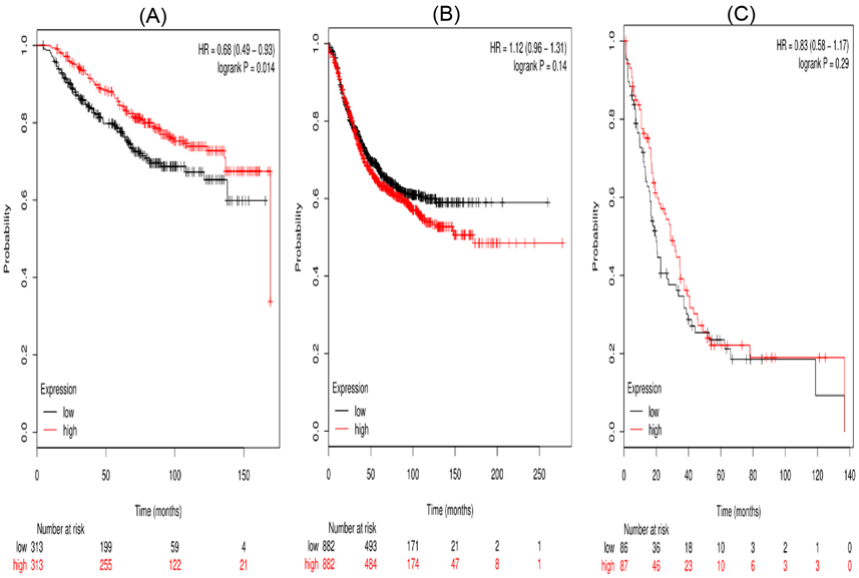
The prognostic value of high STAT3 mRNA expression in breast cancer patients((A) OS curve; (B) RFS curve; (C) PPS curve)

Third, the prognostic significance of *STAT3* mRNA expression in the database was evaluated. Affymetrix IDs for STAT3: 225289_at. High *STAT3* mRNA level showed a significant association with OS (HR =0.68 (0.49–0.93), *P*=0.014) in breast cancer patients. But there was no significant association between high mRNA expression of STAT3 and RFS (HR =1.12 (0.96–1.31), *P*=0.14) or PPS (HR =0.83 (0.58–1.17), *P*=0.29) (Figure 3).

**Figure 4:**
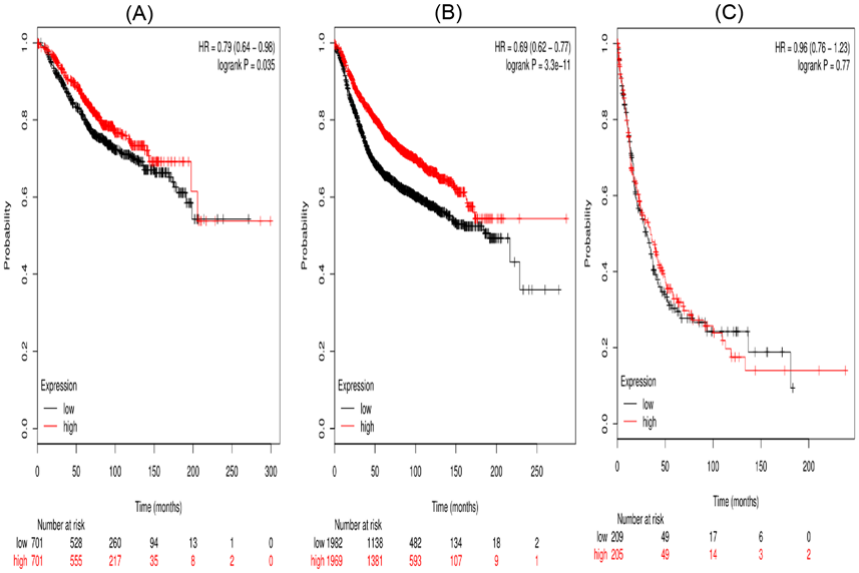
The prognostic value of high STAT4 mRNA expression in breast cancer patients((A) OS curve; (B) RFS curve; (C) PPS curve)

Figure 4 showed the prognostic value of STAT4 in the database for OS, RFS, and PPS in breast cancer. Affymetrix IDs for STAT4: 206118_at. Overexpression of *STAT4* mRNA was significantly associated with a favorable OS (HR =0.79 (0.64–0.98), *P*=0.035) (Figure 4A), RPS (HR =0.69 (0.62–0.77), *P*<0.001) for breast cancer patients. However, high *STAT4* mRNA expression was uncorrelated with PPS (HR =0.96 (0.76–1.23), *P*=0.77) (Figure 4C).

**Figure 5:**
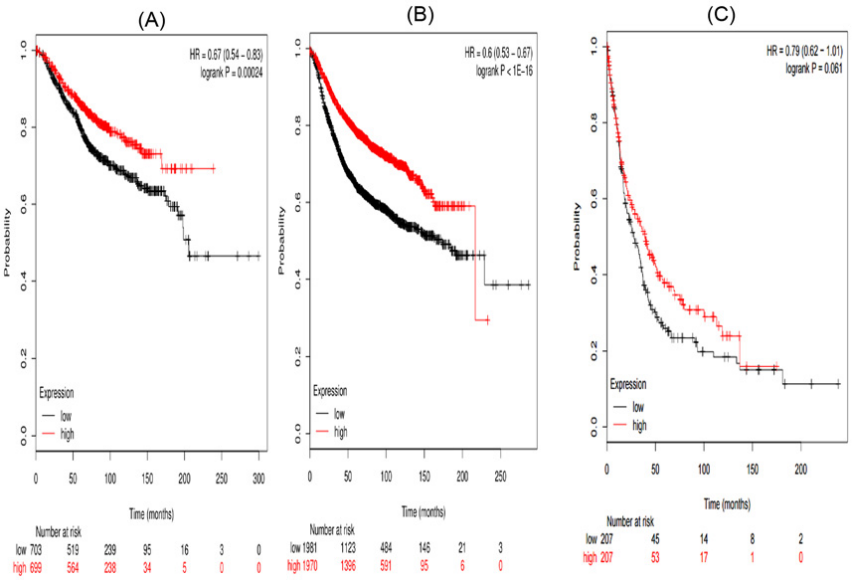
The prognostic value of high STAT5a mRNA expression in breast cancer patients ((A) OS curve; (B) RFS curve; (C) PPS curve)

**Figure 6:**
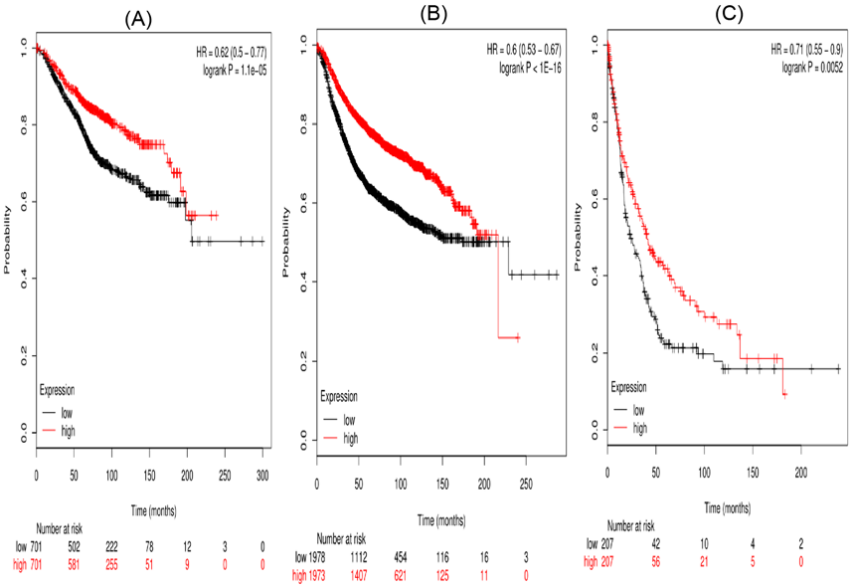
The prognostic value of high STAT5b mRNA expression in breast cancer patients((A) OS curve; (B) RFS curve; (C) PPS curve)

Figures 5 and 6 suggested the prognostic significance of *STAT5a* and *STAT5b*, respectively. Affymetrix IDs were as following: 203010_at (*STAT5a*) and 212549_at (*STAT5b*). Both high *STAT5a* and *STAT5b* mRNA expression are associated with a favorable OS and PFS in breast cancer patients (Figures 5A,B and 6A,B). In addition, STAT5b is significantly associated with a favorable PPS in breast cancer patients (HR =0.71 (0.55–0.9), *P*=0.0052) (Figure 6C).

**Figure 7:**
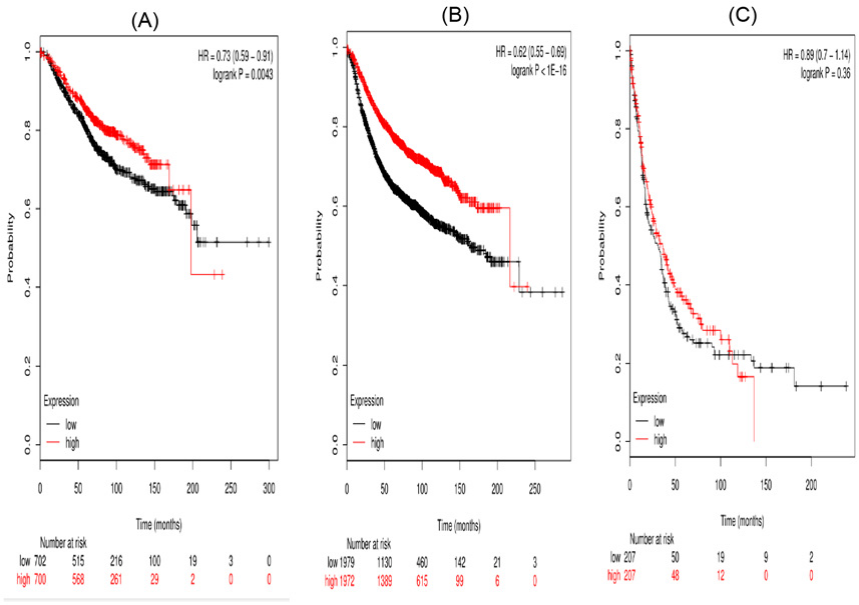
The prognostic value of high STAT6 mRNA expression in breast cancer patients((A) OS curve; (B) RFS curve; (C) PPS curve)

Figure 7 presented the prognostic significance of *STAT6* mRNA expression. Affymetrix IDs for STAT6: 201331_s_at. Overexpression of *STAT6* mRNA were significantly related to favorable OS (HR =0.73 (0.59–0.91), *P*=0.043 and PFS (HR =0.62 (0.55–0.69), *P*<0.001) for all the breast cancer patients. Nevertheless, high STAT6 showed no effect on PPS in cancer patients, HR =0.89 (0.7–1.14), *P*=0.36 (Figure 7C).

In order to further investigate the association of individual STAT with other clinicopathological characteristics, we explored the correlation of individual STATs with pathological grades, ER status, HER2 status, and TP53 mutation of breast cancer patients. We found that except for STAT2 and STAT6, high mRNA expression of STATs was related to a favorable prognosis especially for high pathological grade (II/III) (Table 1). With regard to ER and HER2 status, the association between high mRNA expression of STAT5 and ER status was significant, but association between other high *STATs* mRNA expression and ER status was not significant (Table 2). The associations between *STATs* mRNA and HER2 status was not significant except STAT4 and HER2 negative (HR =0.31 (0.1–0.92), *P*=0.026) (Table 3). In addition, the results indicated that the correlation of STAT1 with TP53 mutation was significant (HR =0.31 (0.12–0.76), *P*=0.072), while the correlation of other STATs with TP53 mutation was not significant for breast cancer patients (Table 4).

**Table 1:** Correlation of *STATs* mRNA high expression with OS in different pathological grades of breast cancer

STATs	Pathological grade	Cases	HR (95% CI)	*P*-value
STAT1	I	56	1.24 (0.5–3.12)	0.6423
	II	325	1.44 (0.93–2.21)	0.0972
	III	1024	0.55 (0.39–0.77)	0.00041
STAT2	I	378	1.91 (0.59–6.16)	0.2686
	II	1077	1.33 (0.85–2.08)	0.2146
	III	1090	0.93 (0.56–1.54)	0.7653
STAT3	I	378	1.91 (0.17–21.57)	0.5967
	II	1077	0.42 (0.13–1.41)	0.1491
	III	1090	0.45 (0.27–0.77)	0.0027
STAT4	I	378	0.58 (0.23–1.47)	0.2456
	II	1077	0.9 (0.59–1.39)	0.6489
	III	1090	0.66 (0.47–0.92)	0.00134
STAT5a	I	56	0.53 (0.21–1.38)	0.1886
	II	325	0.71 (0.46–1.1)	0.1229
	III	1024	0.72 (0.52–1)	0.05222
STAT5b	I	56	0.68 (0.26–1.78)	0.4355
	II	325	0.6 (0.38–0.93)	0.0219
	III	1024	0.92 (0.66–1.28)	0.6183
STAT6	I	56	0.74(0.29–1.9)	0.5278
	II	325	0.89 (0.58–1.37)	0.6017
	III	1024	0.92 (0.66–1.28)	0.6231

**Table 2:** Correlation of *STATs* mRNA high expression with OS in ER status of breast cancer patients

STATs	ER status	Cases	HR (95% CI)	*P*-value
STAT1	Positive	2565	1.4 (0.98–1.99)	0.065
	Negative	1214	0.85 (0.54–1.34)	0.48
STAT2	Positive	2565	1.24 (0.58–2.65)	0.58
	Negative	1214	1.1 (0.54–2.23)	0.79
STAT3	Positive	2565	0.55 (0.25–1.18)	0.12
	Negative	1214	0.79 (0.57–1.1)	0.17
STAT4	Positive	2565	0.86 (0.6–1.23)	0.4
	Negative	1214	0.64 (0.4–1.02)	0.06
TAT5a	Positive	2565	0.63 (0.43–0.91)	0.012
	Negative	1214	0.57 (0.35–0.92)	0.02
STAT5b	Positive	2565	0.79 (0.55–1.12)	0.18
	Negative	1214	0.96 (0.61–1.52)	0.87
STAT6	Positive	2565	0.84 (0.58–1.2)	0.33
	Negative	1214	0.65 (0.41–1.04)	0.071

**Table 3:** Correlation of *STATs* mRNA high expression with OS in HER2 status of breast cancer patients

STATs	HER2 status	Cases	HR (95% CI)	*P*-value
STAT1	Positive	416	0.83 (0.41–1.68)	0.6
	Negative	1456	0.99 (0.42–2.35)	0.98
STAT2	Positive	416	1.32 (0.4–4.36)	0.65
	Negative	1456	0.94 (0.33–2.7)	0.91
STAT3	Positive	416	1 (0.3–3.29)	1
	Negative	1456	0.74 (0.26–2.12)	0.57
STAT4	Positive	416	0.75 (0.37–1.52)	0.42
	Negative	1456	0.31 (0.1–0.92)	0.026
STAT5a	Positive	416	0.56 (0.27–1.17)	0.12
	Negative	1456	0.72 (0.3–1.75)	0.47
STAT5b	Positive	416	1.22 (0.6–2.49)	0.58
	Negative	1456	0.73 (0.29–1.84)	0.5
STAT6	Positive	416	0.95 (0.47–1.9)	0.88
	Negative	1456	0.61 (0.25–1.52)	0.29

**Table 4:** Correlation of *STATs* mRNA high expression with OS in TP53 mutation status of breast cancer patients

STATs	TP53 mutation	Cases	HR (95% CI)	*P*-value
STAT1	No	363	1.05 (0.55–2.01)	0.87
	Yes	232	0.31 (0.12–0.76)	0.0072
STAT2	No	363	Not available	Not available
	Yes	232	0.5 (0.12–1.99)	0.31
STAT3	No	363	Not available	Not available
	Yes	232	0.69 (0.18–2.63)	0.59
STAT4	No	363	0.66 (0.34–1.27)	0.21
	Yes	232	0.6 (0.28–1.3)	0.19
STAT5a	No	363	0.53 (0.27–1.02)	0.054
	Yes	232	0.63 (0.28–1.38)	0.24
STAT5b	No	363	0.4 (0.2–0.8)	0.0072
	Yes	232	0.89 (0.4–1.96)	0.76
STAT6	No	363	0.73 (0.38–1.39)	0.33
	Yes	232	0.93 (0.42–2.06)	0.86

## Discussion

In the present study, KM plotter database was used to comprehensively assess the prognostic value of seven STAT members in breast cancer patients. Our results indicated that STAT3, STAT4, STAT5a, STAT5b, and STAT6 were significantly associated with favorable OS in breast cancer patients, especially for high pathological grade patients. The possible mechanisms may be that tumors that activate these pathways are less aggressive than tumors that progress even in the absence of STAT activation. Alternatively, it may be that STAT plays a role as a tumor suppressor protein [17].However, the specific mechanism of the action is not yet clear, and it still needs to be further clarified. STAT2, STAT4, STAT5a, STAT5b, and STAT6 had significantly favorable effect on RFS of breast cancer patients, but STAT1 had significant worse effect on RFS; STAT5b had significant favorable effect on PPS, while, STAT2 had significant worse effect on PPS for breast cancer patients.

STAT1 has been considered as a growth suppressor based on its role as a pro-apoptotic and antiproliferative molecule [18]. However, the results are still controversial. Previous study reported that increased STAT1 expression and high STAT1 activation (p-STAT1 protein levels) were related to a favorable prognosis in breast cancer [19]. While, Sheen-Chen et al. [20] reported that STAT1 has no association with OS in breast cancer. Our results show that high *STAT1* mRNA expression is not significant with OS in breast cancer patients (HR =0.94 (0.76–1.17), *P*=0.58), but significant with high pathological grade (HR =0.55 (0.39–0.77), *P*=0.00041), which indicated that STAT1 can be a prognostic marker for higher stage of breast cancer patients.

The studies on the function of STAT2 in malignancies were not many, in the present study we found that high *STAT2* mRNA level was not significantly associated with OS. However, high *STAT2* mRNA level was significant associated with favorable PFS (HR =0.55 (0.47–0.64)) and worse PPS (HR =1.45 (1.01–2.08)).

STAT3 is an oncogenic transcription factor and is considered as an emerging target for cancer therapy [21]. It is mainly through dimerization upon phosphorylation at tyrosine residues and translocation to cell nuclei [22]. STAT3 signaling is constitutively activated in various malignant human cancers and participating in multiple cellular progress as well as tumorigenesis [23]. Targetting STAT3 in cancer treatment has shown therapeutic benefits in both preclinical and clinical studies [24–26]. In addition, a cohort study supported that STAT3 overexpression in node-negative breast cancer is associated with a better prognosis [17], which was consistent with our results that high *STAT3* mRNA level was significantly associated with favorable OS (HR =0.68 (0.49–0.93)). STAT3 may act as a promising biomarker for breast cancer.

Similar to STAT2, the evidence of STAT4 expression in breast cancer patients was limited [27]. Our results indicated that high *STAT4* mRNA expression was significantly associated with favorable OS and RFS in breast cancer patients. More studies are needed to validate our results.

For STAT5, there are two homologous isoforms (STAT5a and STAT5b) [28]. Study proved that under inactive state, the STAT5 protein is present in the cytoplasm of quiescent cells in normal breast tissues. However, in breast cancer cells, STAT5 is continuously activated and translocated into the nucleus [29]. A number of studies reported that STAT5 genes could predict a better survival in malignancies, such as lung cancer [30], cervical cancer [31], breast cancer [32]. Our results suggest that high mRNA expression of STAT5a and STAT5b is significantly associated with favorable OS, which is consistent with previous findings [28]. In addition, we found that high mRNA expression STAT5a and STAT5b is significantly associated with RFS in breast cancer patients, implying that STAT5 may be an important promising biomarker for breast cancer.

Up to now, the studies on STAT6 are mainly focussed on breast cell lines [33,34]. Han et al. [35] reported that the nuclear STAT6 being positive is a helpful and highly sensitive marker in diagnosis of solitary fibrous tumors/hemangiopericytomas. And Li et al. [36] reported that high STAT6 expression was correlated to a better OS of ovarian cancer patients. Our results indicate that high mRNA expression of STAT6 is significantly associated with OS and RFS in breast cancer patients.

As we know, breast cancer is a complicated disease and its prognosis may be related to different factors [37]; and breast cancer is divided into different subtypes [38,39]. We explored the prognostic values of individual STATs under different ER status and HER2 status. Our results suggested that high *STAT5* mRNA expression was correlated with ER no matter ER positive or negative (Table 2) and only high *STAT4* mRNA expression was associated with HER2 negative status (HR =0.31 (0.1–0.92), *P*=0.026) (Table 3). While, Yang et al. [40] found that hormone therapy (Tamoxifen) failed to improve the OS in patients presenting with increased protein inhibitor of activated STAT3 expression, which was consistent with our result. And research has shown that up to 70% of patients who receive adjuvant HER2-targetted therapy (Trastuzumab) after chemotherapy experience disease progression due to both *de novo* and acquired resistance, and acquired resistance [41,42]. And the HER2 status in breast patients may vary during disease progression [43,44]. In addition, the underlining mechanism by which STAT is activated or depressed by different hormones and distinct STAT factors’ functions in breast cancer has not been well elucidated. Thus, a large number of experiments are still needed to prove that.

In addition, we investigated the prognostic values of individual STATs in TP53 mutation. Our results show that high *STAT1* mRNA expression in TP53 mutated and high *STAT5b* mRNA expression in wild-type suggested a favorable OS. As the number of breast cancer patients in TP53 mutation is limited, more studies with large sample size are warranted to predict the prognostic value of STATs family.

## Conclusion

In conclusion, our results suggest that high mRNA expression of all the individual STATs, except STAT1 and STAT2, are significantly associated with favorable OS in breast cancer patients, especially for the high pathological grade. In addition, our results show that high mRNA expression of STAT1 is significantly associated with worse RFS for the breast cancer patients; all the other individual STATs except STAT3 are significantly associated with better RFS in breast cancer patients; only high *STAT5b* mRNA expression is significantly associated with better PPS for the breast cancer patients. And we also found that high *STAT5* mRNA expression indicates a favorable prognosis no matter under ER positive or negative status; high *STAT4* mRNA expression suggests a favorable prognosis under HER2 negative status. Our results indicate that STATs family play a significantly prognostic role in breast cancer patients and individual STATs, except STAT1 and STAT2, and may be a favorable prognostic biomarker in breast cancer. Owing to limited data, the relationship between high individual *STATs* mRNA expression and the prognosis of breast cancer needs further verification.

## Abbreviations

ER: estrogen receptor
HER2: human epidermal growth factor receptor 2
HR: hazard ratio
KM plotter: Kaplan–Meier plotter
OS: overall survival
PFS: progression-free survival
PPS: post-progression survival
STAT: signal transducer and activator of transcription
TP53: tumor protein P53
